# Dynamic of a Delayed Predator-Prey Model with Application to Network’ Users’ Data Forwarding

**DOI:** 10.1038/s41598-019-48975-8

**Published:** 2019-08-29

**Authors:** Yaming Zhang, Yaya Hamadou Koura, Yanyuan Su

**Affiliations:** School of Economics and Management, Yanshan University, Qinhuangdao, 066004 China

**Keywords:** Applied mathematics, Complex networks

## Abstract

In most situations where entities interact by sharing limited resources, controlling populations’ density is crucial to maintain ecosystem sustainability. This is the case in a predator-prey type interaction when predator survival relies on its ability to harvest and consume resources. In this article, we analyzed a modified predator-prey model based on Rosenzweig-MacArthur characterized by a delayed conversion of prey into resources and applied the proposed model to network users’ data forwarding at a bottleneck node. We discuss system fixed points behaviour and prove that delaying the handling time has a significant impact on the dynamic of interaction and system bifurcates, exhibits chaotic behaviour and is highly responsive to small perturbations.

## Introduction

In applied engineering and complex system sciences, mathematical models that display deterministic chaotic dynamical behaviour are of interest. For such systems, it is necessary to analyze stability of some given nonhyperbolic trajectories around equilibrium points and determine whether these systems exhibit rich dynamic or not. Examining bifurcation, especially the supercritical ones, is very common in population dynamics, as one can determine a set of periodic solutions that may lead to system stabilization or to chaos^1–4^. Rosenzweig-MacArthur predator-prey model is one of such model that presents the advantage of being simple and yet exhibits very rich dynamics. It is mostly used to study bifurcation and chaotic behaviour in predator-prey interactions. Adding Holling Type II terms in the modified version allows better control of populations’ density and handling time of the predator, which is important to control its growth whereas the classical model assumes searches are random and that predator search rate and handling time are constant. Predator growth is proportional to its prey population size or density. Holling Type II supposes maximum mortality of predator at low prey density^5–9^. In most of ecosystems, predator searching and handling efficiency is strongly dependent on prey density or resources availability. In our approach, we separate predator searching time and handling time by introducing a delay parameter in the differential equations. In nature, it is admitted that most of interactions occur in delayed or discrete fashion, as both predator and prey act stochastically in consuming available resources. This can be applied to network users that share bandwidth and resources at a bottleneck node or a leaky bucket set up to monitor traffic flows for example. If we consider network users’ behaviour to be stochastic and the accommodating segment to have limited buffering space then, in rush hours, when users interact intensively, forwarding generated data packets can be assimilated to a predator-prey type interaction with limited resources characteristics. Analyzing network packets forwarding to depict the performance of a particular node or segment is important in understanding users’ behaviour impact on the overall performance of the network during peak hours for informing decisions made locally at certain given segments^10–13^.

Many authors have studied the dynamic of classical Rosenzweig-MacArthur predator-prey model and there are numerous published articles on this subject. Particularly, one can find several modified Rosenzweig-MacArthur models studied in the related literature^3,6,14–20^. In our approach, instead of modelling the underlying relationship between network users using a classical predator-prey competitive system as in the previous published article^21^, we have chosen a Rosenzweig-MacArthur type model for its accuracy in capturing density dependency phenomenon and sensitivity to small perturbations. While a classical competitive model focuses mainly on the outcome of competition, this can be limiting for tuning or adjusting parameters. If we observe the underlying relationship in this particular situation at a leaky bucket for instance, where all packets are mixed before being moved to the output link, it becomes clear that a ratio-dependant type model like Rosenzweig-MacArthur predator-prey system is a logical choice. This model not only emphasises predator survival dependency on prey population size via the functional response, but also offers more possibilities in adjusting parameters during low network traffic. In real world or real complex networks this may be necessary as Internet network has become ubiquitous and system segment, at peak hours could forward heterogeneous data packets with variable QoS (quality of service) requirements such as voice data, multimedia data etc^22^.

In this paper, we present a predator-prey model based on Rosenzweig-MacArthur in which predator handling time is delayed to allow prey to grow faster consuming all available resources. We assimilate this phenomenon to priority given to data packets flow with higher quality of service requirements, knowing that most of routers and switchers are configured to perform “Best-Effort Traffic” by forwarding all packets in hand. Their limiting capacity in terms of memory space or buffers has a significant influence on the amount of packets to process per unit time. In TCP/IP, window size and allocated resources in transferring packets could be less or over estimated in the first round in most of configurations^23^. As system is neutral in handling and forwarding packets and users’ behaviour is random, it is logical to model this particular type of interaction using a modified Rosenzweig-MacArthur predator-prey system.

We performed qualitative analysis of the proposed model and determined stability and bifurcation conditions at system fixed points. We applied theoretical results to a network bottleneck node where users are sending traffic by considering high priority users as prey and low priority users as predators. Results of the numerical simulation suggest adjusting delay and priority in the congestion control mechanism to avoid latency and poor quality of service at rush hours.

## Preliminary

Letting *P* be prey density at time *t*, *K* the carrying capacity of the environment or segment buffering capacity, and assuming prey growth density over time obeys logistic pattern $P(1-P/K)$. If we consider predator handling rate is governed by Holling type II functional response of the form $Q/(a-P)$, where *Q* is the predator density at time *t*, we can write

1 $$\frac{dP}{dt}=P[r(1-\frac{P}{K})-\frac{Q}{a+P}],$$

where *dP* is the first order derivative, representing density variation of the prey in function of time, *r* is its intrinsic growth factor in the absence of the predator. This factor represent ratio of packets generated per time unit in respect to the ratio of prey packets present in the system.

$Q/(a+P)$ is the functional response representing predator handling. *a* is a positive parameter denoting conversion rate of prey into resources.

Similarly, for the predator equation, we have

2 $$\frac{dQ}{dt}=Q[\beta Q+\frac{P(t-\tau )}{a+P}],$$

where *dQ* is the first order derivative representing predator density variation over time, β is its intrinsic growth factor in the absence of the prey. *τ* is the delay factor and *t* − *τ* is the delaying terms denoting the fact that predator needs time to assimilate captured prey depending on system configuration. $P(t-\tau )/(a+P)$ represents the functional response which corresponds to the assimilation efficiency. It is clear that predator growth over time is submitted to delay needed for processing predated preys and by the value of parameter *a*. A larger population of prey will ends up having a negative impact on predator population size, as the functional response will tend to one. Furthermore, in the absence of prey, as resources are limited and predator cannot grow exponentially, we assume the maximum population size the system can accommodate is *K* the carrying capacity (in terms of value to reach).

## Interactive Model

Consider following assumptions hold:

(i) System is giving priority to the prey in resources consumption.
(ii) The predator has access to resources only when there is room in the buffers and by handling and converting captured preys.
(iii) During time interval $[t-1,t]$, prey and predator have constant growth rate for analysis purpose.
(iv) *P* density has logistic growth in respect to carrying capacity.
(v) Prey decreases density in the presence of predator or when meeting.
(vi) In the absence of prey, *K* is the maximum value to reach for *Q* (carrying capacity).

The interaction model can be written as follows:

3 $$\begin{array}{c}\{\begin{array}{c}\frac{dP}{dt}=P[r(1-\frac{P}{K})-\frac{Q}{a+P}-\theta ],\\ \frac{dQ}{dt}=Q[\beta Q+\frac{P(t-\tau )}{a+P}-c],\end{array}\\ K=P(t)+Q(t);\\ P(t),Q(t)\le K,\\ P(0),Q(0)\ge 0;\\ \theta ,c\in ]\mathrm{0...1}[;\\ r,K,a,\beta ,\tau  > 0.\end{array}$$

where *r* and *β* represent *P* and *Q* respective growth factor. *a* is the conversion rate per capita of prey into resources for the predator. *θ* and *c* are the decay factors per unit time of the prey and the predator respectively, when density decreases for any reason related to system state and other factors. *τ* represents the delay needed to process queuing preys’ packets due to predator consumption of resources (buffering and computing). *K* is segment carrying capacity representing the total amount of packets system can accommodate. At any time during the interaction, prey population is kept under the value of *K*, which is the maximum value to reach for *P* in respect to logistic equation.

*P* increases density only if $dP/dt > 0$. Knowing that $K > P > 0;r,a,\theta  > 0,$ when time *t*_*i*_, $i=\mathrm{1...}\infty$, *P* has to be kept smaller enough to allow prey to thrive. Similarly for the predator equation, *Q* increases density only if $dQ/dt > 0$. Knowing $K > Q > 0;\beta ,c,\tau  > 0,$ when time *t*_*i*_, $i=\mathrm{1...}\infty$, *c* and *τ* have to be chosen smaller enough to allow predator to thrive until reaching the value of *K*.

System (3) zero growth isoclines is determined by solving

4 $$\begin{array}{c}r(1-P/K)-Q/(a+P)-\theta =0,\\ \beta Q+P(t-\tau )/(a+P)-c=0.\end{array}$$

We have

5 $$\begin{array}{c}f(P,Q)=-\,r{P}^{2}+(rK-ar)P-KPQ-K(1+a)Q+arK=0,\\ g(P,Q)=P[(t-\tau )-c]+\beta PQ+a\beta Q-ac=0.\end{array}$$

**Figure 1:**
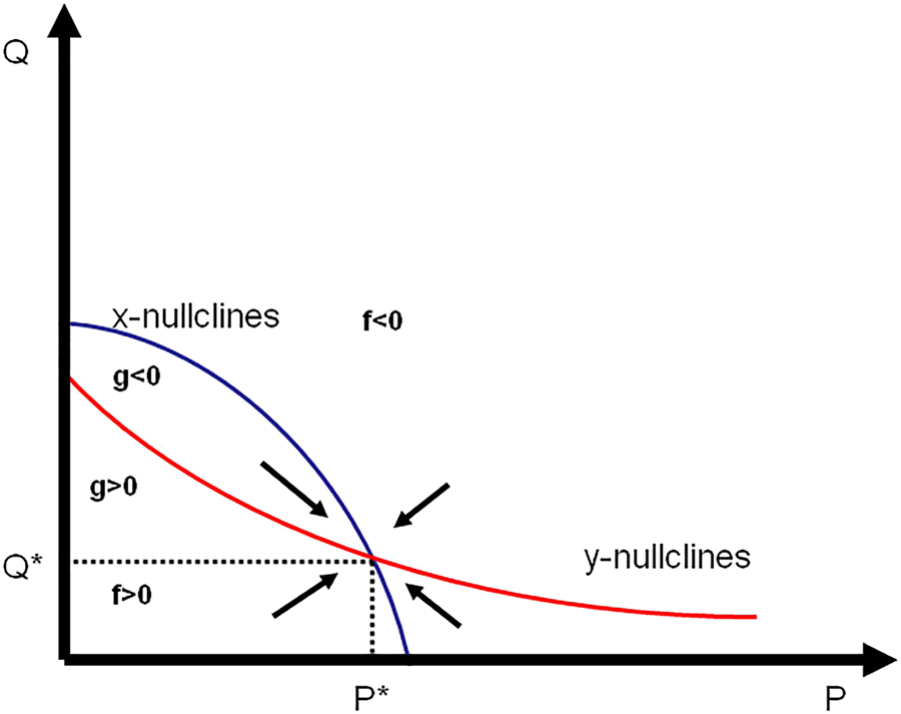
Phase diagram near the unique positive equilibrium point when predator and prey interact. This point, intersection of the x-isoclines (in blue) and y-isoclines (in red) is attracting all close trajectories.

This implies that system could have more than one positive solution in the first quadrant depending on parameters value and the initials. By solving (5), we have found that in some cases, system admits two solutions in int ${{\mathbb{R}}}_{+}^{2}$, and any of them could be stable depending on parameters value and system state. The most attractive case is when system admits only one positive solution in the positive quadrant corresponding to the case both predator and prey increase population density as shown in Fig. 1.

If we consider *f* and *g* as continuous and differentiable functions in ${{\mathbb{R}}}_{+}^{2}$ then, based on Poincaré-Bendixson criterion, we can write, for any $E=({P}^{\ast };{Q}^{\ast })$ positive equilibrium point of the system (3):

6 $$div(f,g)=\nabla \cdot (f,g)=\frac{\partial f}{\partial P}+\frac{\partial g}{\partial Q}=r+2\beta {Q}^{\ast }+\frac{{P}^{\ast }(t-\tau )}{K}-(\frac{2r{P}^{\ast }}{K}+\frac{{Q}^{\ast }}{a+{P}^{\ast }}+\theta )$$

Letting

7 $$\begin{array}{c}{{\rm{\Phi }}}_{1}=r+2\beta {Q}^{\ast } > 0,\\ {{\rm{\Phi }}}_{2}=(\frac{2r{P}^{\ast }}{K}+\frac{{Q}^{\ast }}{a+{P}^{\ast }}+\theta )-\frac{{P}^{\ast }(t-\tau )}{K} > 0,t < \tau ,\end{array}$$

we have

8 $$\frac{\partial f}{\partial P}+\frac{\partial g}{\partial Q}={{\rm{\Phi }}}_{1}-{{\rm{\Phi }}}_{2}\ne 0.$$

We can conclude that there exists at least one periodic orbit depending on parameters value.

If ${{\rm{\Phi }}}_{1} > {{\rm{\Phi }}}_{2}$ then, system (3) has a limit cycle in ${{\mathbb{R}}}_{+}^{2}$.

If ${{\rm{\Phi }}}_{1} < {{\rm{\Phi }}}_{2}$ then, system (3) has no limit cycle in ${{\mathbb{R}}}_{+}^{2}$.

### Model equilibrium points

Letting

$$\begin{array}{c}{m}_{1}=r-\theta -1 > 0,\\ {m}_{2}=c+1 > 0,\end{array}$$

$${m}_{3}=1/(a+{P}^{\ast }) > 0,$$

where

$$\mathop{\mathrm{lim}}\limits_{P\to \infty }{P}^{\ast }\to \infty ,{m}_{3}\to 0.$$

It is clear that system (3) admits the following equilibrium points in the positive quadrant: *O* = (0; 0) the origin, $M=({m}_{1}K/r;0)$ on the *x*-axis, when predators are extinct, $N=(0;{m}_{2}/\beta )$ on the *y*-axis, when preys are extinct. In the case predator and prey coexist and interact, we will restrict our analysis on the positive quadrant, assuming, based on Lyapunov theorem, system admits a unique positive equilibrium point expressed as

9 $$\begin{array}{c}E=(\frac{K(\beta {m}_{1}-{m}_{2}{m}_{3})}{r\beta -K(t-\tau ){({m}_{3})}^{2}};\frac{r{m}_{2}-{m}_{1}{m}_{3}K(t-\tau )}{r\beta -K(t-\tau ){({m}_{3})}^{2}}),\\ r\beta  > K(t-\tau ){({m}_{3})}^{2},\\ \beta {m}_{1} > {m}_{2}{m}_{3},\\ r{m}_{2} > {m}_{1}{m}_{3}K(t-\tau ).\end{array}$$

Condition (9) can be satisfied if *t* − *τ* is chosen smaller such that *t* − *τ* → 0, or larger density of the prey such that $P\to \infty$. Similarly, $c\to \pm 0$.

## Model Stability Analysis

System (3) linearized form is given as

10 $$J=(\begin{array}{cc}r-(\frac{2r{P}^{\ast }}{K}+\frac{{Q}^{\ast }}{a+{P}^{\ast }}+\theta ) & \frac{{P}^{\ast }}{a+{P}^{\ast }}\\ \frac{{Q}^{\ast }(t-\tau )}{{(a+{P}^{\ast })}^{2}} & 2\beta {Q}^{\ast }+\frac{{P}^{\ast }(t-\tau )}{K}\end{array})$$

**i –** Evaluated at the origin, we have

11 $$J=(\begin{array}{cc}r-\theta  & 0\\ 0 & 0\end{array})$$

If *r* > *θ* then, the origin point is a nodal unstable saddle source repelling all nearby trajectories. If *r* < *θ* then, the origin point is a nodal stable saddle sink attracting all closer enough trajectories.

Furthermore, the polynomial equation *λ*^2^ = *r* − *θ* gives,

$${\lambda }_{1,2}=\pm \,\sqrt{r-\theta }$$ if $$r > \theta $$,

$${\lambda }_{1,2}=\pm \,i\sqrt{|r-\theta |}$$ if $$r < \theta $$,

$${\lambda }_{1,2}=0$$ if $$r=\theta $$.

**Figure 2:**
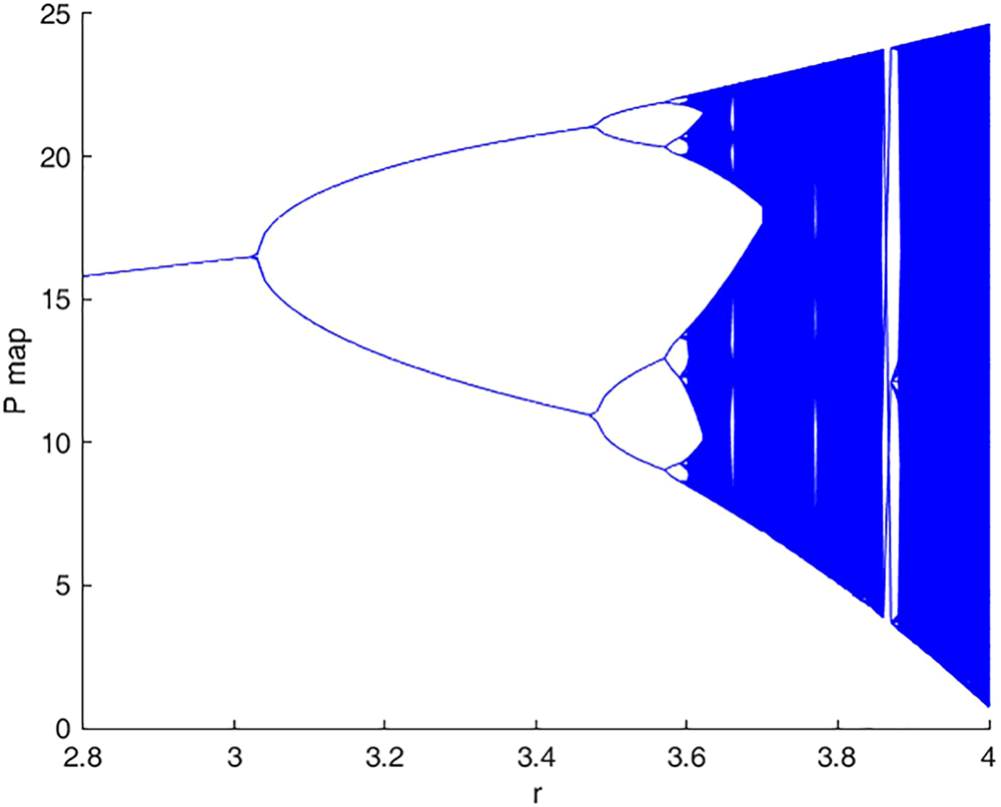
Bifurcation diagram of P map (y-axis) showing changes in system behaviour with *r* covering [2.8, 4] (x-axis) for P(0) = 1.6, K = 25 and θ = 0.03.

In the case the origin is a saddle, we can consider *r* as the bifurcation parameter as in the classical logistic equation. Figure 2 shows how this parameter affects system behavior when its value varies in the given range [2.8, 4]. For each value of *r*, points lying on the stable orbit are plotted. It can be seen that there are four families of periodic orbits.

**ii –** Evaluated at the equilibrium point *M*, we have

12 $$J=(\begin{array}{cc}1-{m}_{1} & \frac{{m}_{1}K}{ar+{m}_{1}K}\\ 0 & \frac{{m}_{1}K(t-\tau )}{ar+{m}_{1}K}\end{array})$$

*M* is asymptotically stable only if $1-{m}_{1} < 1$ and ${m}_{1}K(t-\tau )/(ar+{m}_{1}K) < 1$. This could be satisfied if ${m}_{1} > 0$ and $t-\tau  < (ar+{m}_{1}K)/{m}_{1}K$.

In the case *m*_1_ < 0 or $t-\tau  > (ar+{m}_{1}K)/{m}_{1}K$, the equilibrium point will behave as an unstable saddle node. This point will turn into a source node if *m*_1_ > 0 and $t-\tau  > (ar+{m}_{1}K)/{m}_{1}K$.

Solutions around *M* are nonhyperbolic in the special case when *m*_1_ = 1 and $t-\tau =(ar+{m}_{1}K)/{m}_{1}K$. This happens when *r* − *θ* = 2 and *t* > τ, implying that system stability around *M* on the *x*-axis is weaker; a small perturbation may ends up in significant changes in the dynamic of the interaction between species.

Trace and determinant of the Jacobian matrix are given

13 $$\begin{array}{c}Tr(J)=\frac{(1-{m}_{1})(ar+{m}_{1}K)+{m}_{1}K(t-\tau )}{ar+{m}_{1}K},\\ \det (J)=\frac{(1-{m}_{1}){m}_{1}K(t-\tau )}{ar+{m}_{1}K}.\end{array}$$

System bifurcates at *M* if $Tr(J)=0$ and $\det (J) > 0$, that is $(1-{m}_{1})(ar+{m}_{1}K)={m}_{1}K(\tau -t)$ and $(1-{m}_{1}){m}_{1}K(t-\tau ) > 0.$ This could be satisfied if $\tau  > t$ and ${m}_{1} > 1$.

**iii –** Evaluated at the equilibrium point *N*, we have

14 $$J=(\begin{array}{cc}r-\theta -\frac{{m}_{2}}{\beta {a}^{2}} & 0\\ \frac{{m}_{2}(t-\tau )}{\beta {a}^{2}} & 2{m}_{2}\end{array})$$

System is stable around *N* only if $r-\theta -{m}_{2}/\beta {a}^{2} < 1$ and ${m}_{2} < 0.5$. This implies that the decay factor must have very small value to allow the predator to grow in the absence of the prey, consuming all available resources. This analysis is consistent with the understanding of the situation and previous theoretical assumptions.

If $r-\theta -{m}_{2}/\beta {a}^{2} > 1$ or ${m}_{2} < 0.5$, the equilibrium point *N* will behaves as a saddle node stable or unstable depending on the value and sign of the eigenvalues. If $r-\theta -{m}_{2}/\beta {a}^{2} > 1$ or ${m}_{2} > 0.5$, the equilibrium point *N* will behave as a source node unstable for any value of the eigenvalues. If $r-\theta -{m}_{2}/\beta {a}^{2}={m}_{2}=1$, nearby trajectories are nonhyperbolic and could be perturbated easily to obtain or to loose stability depending on the situation. For this purpose, one needs to determine bifurcation conditions.

Trace and determinant of the Jacobian matrix are given

15 $$\begin{array}{c}Tr(J)=\frac{(r-\theta )\beta {a}^{2}-{m}_{2}+2{m}_{2}\beta {a}^{2}}{\beta {a}^{2}},\\ \det (J)=2{m}_{2}[\frac{(r-\theta )\beta {a}^{2}-{m}_{2}}{\beta {a}^{2}}].\end{array}$$

It follows; system bifurcates at *N* if following condition holds: $r > \theta ;(r-\theta )\beta {a}^{2} > {m}_{2}.$

**iv –** Evaluated at the unique positive equilibrium point *E*, we have

16 $$J=(\begin{array}{cc}r-{m}_{4} & {m}_{5}\\ {m}_{6} & 2\beta {Q}^{\ast }+{m}_{7}\end{array})$$

where

$${m}_{4}=\frac{2r{P}^{\ast }}{K}+\frac{{Q}^{\ast }}{a+{P}^{\ast }}+\theta  > 0,{m}_{5}=\frac{{P}^{\ast }}{a+{P}^{\ast }} > 0,{m}_{5}=\frac{{Q}^{\ast }(t-\tau )}{{(a+{P}^{\ast })}^{2}} < 0,{m}_{7}=\frac{{P}^{\ast }(t-\tau )}{K} < 0.$$

System is stable asymptotically around *E* if and only if $r-{m}_{4} < 1$ and $2\beta {Q}^{\ast }+{m}_{7} < 1$, knowing that Q* and P* are positive real numbers.

System is unstable around the saddle node *E* if and only if $r-{m}_{4} > 1$ or $2\beta {Q}^{\ast }+{m}_{7} > 1$. This point behaves as a source node in the case $r-{m}_{4} > 1$ and $2\beta {Q}^{\ast }+{m}_{7} > 1$.

## Numerical Results

We have conducted numerical simulation to verify the proposed model predictability and validate our theoretical analysis. We examined in the first case system dynamic when prey conversion rate is lower. In the second case, we slightly varied parameters’ values and explored system dynamic impact on the predator-prey interaction. In the last case, we examined the case system admits periodic solutions, bifurcates and becomes chaotic for some given set of values to explore the consistency of the proposed model. Prey is assumed to be network users with high priority in shaping the traffic. Their generated packets will always be accommodated first. If there is no room in the buffers, system will discard any incoming packet. Predator has low priority in the traffic shaping and scheduling. While queuing at the buffer space, corrupted packets could be dropped, decreasing respective species’ population density due to network failure, latency or any event related to the congestion control mechanism.

**Figure 3:**
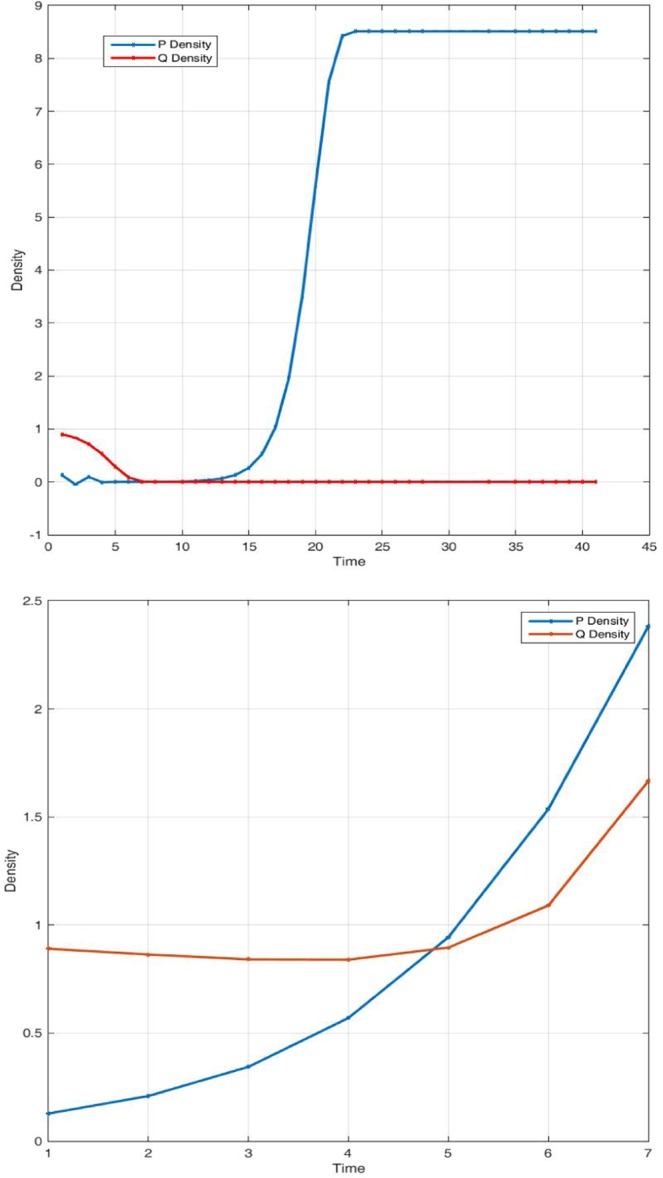
System dynamic over time when the predator dies out (i) suffering from a smaller conversion rate for P(0) = 0.128; Q(0) = 0.9; r = 3.002; a = 0.252; K = 25; θ = 0.98; τ = 1.001232; β = 1.03; c = 0.00030. Coexistence of species is restored in (j), when prey assimilation is instantaneous for P(0) = 0.128; Q(0) = 0.89; r = 3.002; a = 2.252; K = 25; θ = 0.98; τ = 0; β = 1.03; c = 0.00030.

Figure 3i shows interaction dynamic when the low priority user or predator shrinks and vanishes because of its weakness in searching and handling prey. System accommodates only high priority users and their packets occupy all available space in the buffers. When system is configured such that no delay is applied, meaning τ = 0 and priority in accessing resources is cancelled as shown in Fig. 3j, predator and prey coexist peacefully. Both users will access resources at a relative speed depending on parameters values and system state. When a significant delay is applied, predator packets density increases while prey shrinks and dies (i). However, as predator searching becomes harder, it decreases density at high speed. This is consistent with our theoretical analysis. Delaying high priority users’ packets is crucial in maintaining system stability by saving resources that can be allowed to lower priority users. Nevertheless, delay must be smaller enough to allow coexistence of all network users (j).

**Figure 4:**
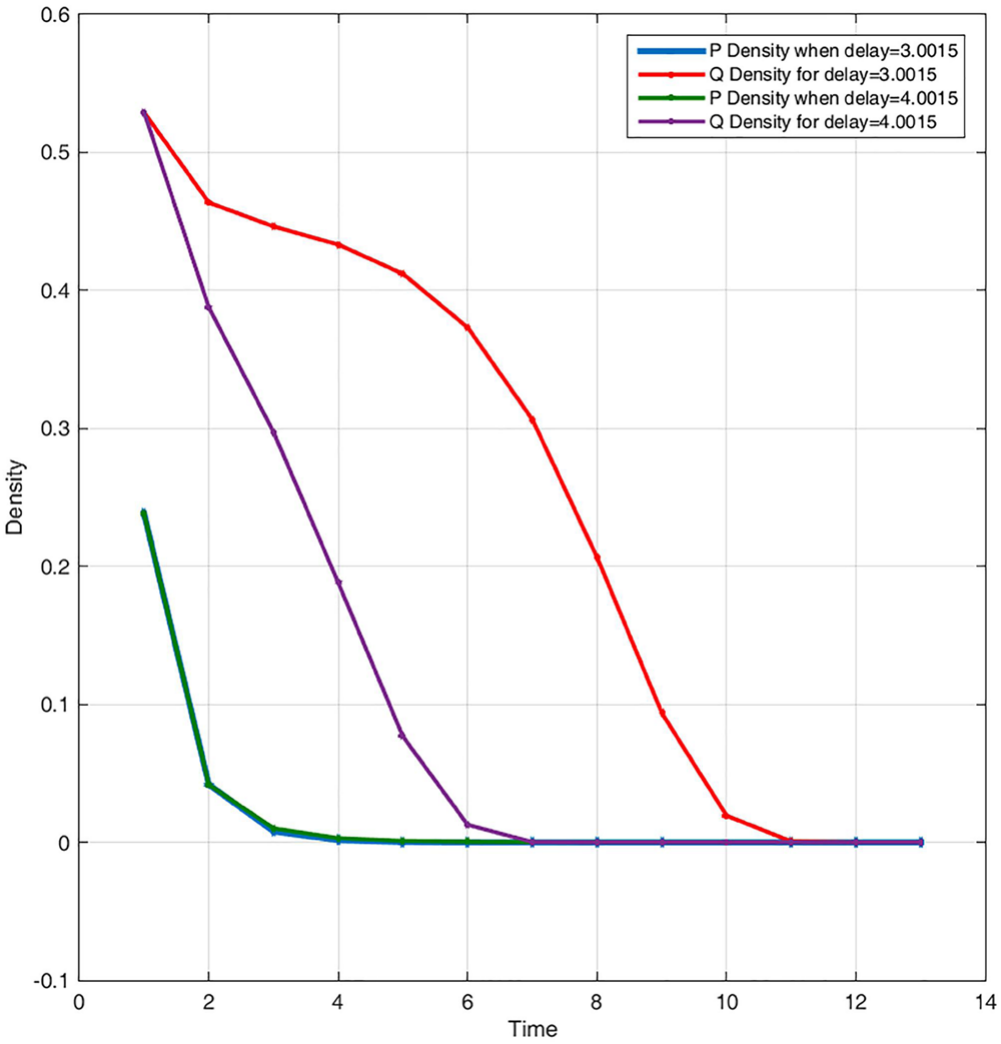
System dynamic over time when the prey suffers much from the delaying effect and shrinks for P(0) = 0.238; Q(0) = 0.529; r = 1.5; a = 1.42; K = 20; θ = 0.98735; τ = 3.0015; β = 2.19995; c = 0.00120.

**Figure 5:**
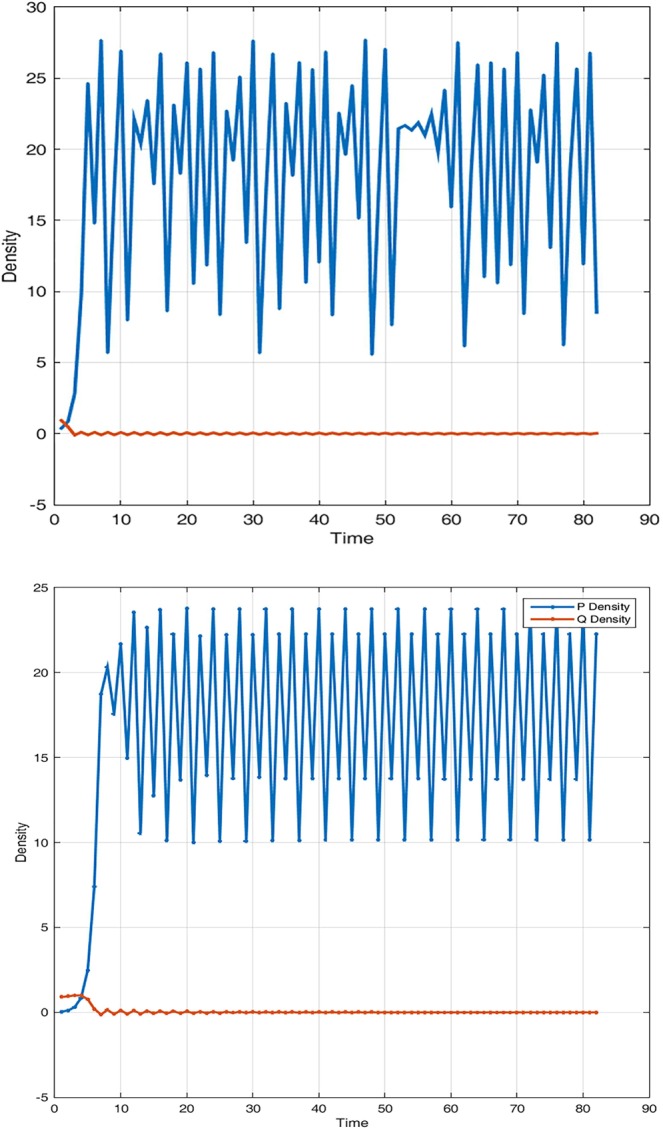
System dynamic over time in the case the predator is extinct and system exhibits chaotic behaviour for (i) P(0) = 0.38; Q(0) = 0.929; r = 3.9; a = 0.25; K = 30; θ = 0.098735; τ = 2.0015; β = 1.1995; c = 0.00120. System regains stability when we vary initials and the conversion rate parameters in (j) for P(0) = 0.38; Q(0) = 0.9; r = 3.9; a = 1.25; K = 30; θ = 0.38735; τ = 2.0015; β = 1.1995; c = 0.0010.

**Figure 6:**
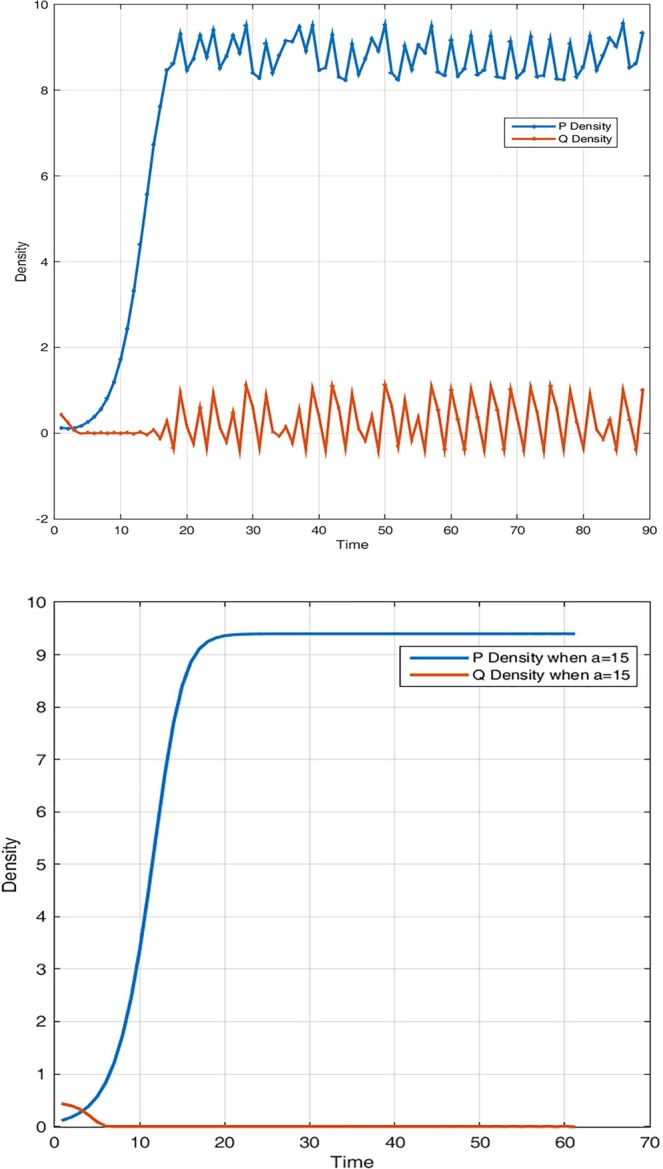
System dynamic over time when both predator and prey exhibit chaotic behaviour (unstable periodic solutions) in (i) for P(0) = 0.1238; Q(0) = 0.429; r = 1.6; a = 0.55; K = 30;θ = 0.098735; τ = 3.0015;β = 2.19995;c = 0.00120 and regains stability in (j) for a larger conversion rate a = 15.

Figure 4 displays system behaviour when lower priority users are extinct. System admits stable periodic solutions when the stability condition is satisfied. When we choose different delay parameter values [3.0015, 4.0015], only the predator dynamic is impacted. When converting resources needs more time, lower priority users’ packets queuing delay variation will negatively affect concerned network users. In Fig. 5i,j, for the same delay parameter, when we vary the initials and the conversion rate of prey into resources, system bifurcates and exhibits chaotic behaviour as shown in Fig. 5i and regains stability as in Fig. 5j. Users’ packets density variation over time has no significant correlation when chaos appears. This is consistent with the understanding of the real situation in that, increasing accommodating segment buffering capacity could lead to buffer bloat issues. In Fig. 6i,j, when we vary the conversion rate parameter [0.55, 15], keeping constant all other parameters, system crosses the bifurcation line passing from chaos to stability.

## Conclusion

We have presented a modified Rosenzweig-MacArthur model with delay in predator assimilation of converted prey. We showed that the proposed model has rich dynamical behaviour and is consistent with the continuous time classical two-dimensional Rosenzweig-MacArthur model where bifurcation and chaos can be obtained easily at the nonhyperbolic fixed points of the system. The model has been proven useful in analyzing interaction occurring at a bottleneck node or leaky bucket when users with different priority send traffic during rush hours. Numerical simulation performed validated our theoretical analysis and suggests adopting hybrid priority and scheduling in the congestion control mechanism to maintain system stability and guarantee QoS.

## References

[1] Hassard, B. D., Kazarinoff, N. D. & Wan, Y. H. Theory and Applications of Hopf Bifurcation, London Mathematical Society Lecture Note Series 41, Cambridge University Press, Cambridge (1981).

[2] Bélair J, Campbell SA (1994). Stability and bifurcations of equilibrium in a multiple-delayed differential equation,. SIAM J. Appl. Math..

[3] Wiggins Stephen (1990). Introduction to Applied Nonlinear Dynamical Systems and Chaos.

[4] Almanza-Vasquez E, Ortiz-Ortiz R-D, Marín AM, Almanza E (2015). Bifurcations in the Dynamics of Rosenzweig-MacArthur Predator-Prey Model Considering Saturated Refuge for the Preys. Applied Mathematical Sciences.

[5] Miller DA, Grand JB, Fondell TF, Anthony M (2006). Predator-Prey Functional Response and Prey Survival: Direct and Indirect Interactions Affecting a Marked Prey Population. J. Animal Ecol..

[6] Canate E, Fong W, Severiche C, Marrugo Y, Jaimes J (2018). Model dynamics of Rosenzweig- MacArthur considering the proportional refuge function to the number of dams. Contemporary Engineering Sciences.

[7] Rosenzweig M, MacArthur R (1963). Graphical representation and stability conditions of predator-prey interaction. American Naturalist.

[8] Xiao D, Zhang Z (2003). On the uniqueness and nonexistence of limit cycles for predator-prey systems. Nonlinearity.

[9] Vlastimil K, Jan E (2006). The Effect of Holling Type II Functional Response on Apparent Competition. Theoretical Population Biology.

[10] Mohamed, S. A. E. A. & Abdelmoty, A. I. Spatiotemporal Analysis of User-Generated Data on the Social Web, (Cardiff University), Cardiff CF24 3AA (UK, 2012).

[11] Dowdy, L. W., Rosti, E., Serazzi, G. & Smirni, E. Scheduling Issues in High Performance Computing, *Performance Evaluation Review* (1999).

[12] Thorenoor, S. G. Dynamic Routing Protocol Implementation Decision between EIGRP, OSPF and RIP Based on Technical Background Using OPNET Modeler, pp. 191–195. ISBN: 978-1-4244-6962-8.

[13] Salonidis, T., Garetto, M., Saha, A. & Knightly, E. Identifying High Throughput Paths in 802.11 Mesh Networks: A Model-Based Approach, *IEEE Intl Conference of Network Protocols(ICNP)*, pp. 21–30 (2007).

[14] Wasike A, Bonga’ng’a S, Lawi G, Nyukuri M, Predator-Prey A (2014). Model with a Time Lag in the Migration. Applied Mathematical Sciences.

[15] Cheng K (1981). Uniqueness of a limit cycle for a predator-prey system. SIAM J. Math. Anal..

[16] Neubert MG, Klepac P, van den Driessche P (2002). Stabilizing Dispersal Delays in Predator-Prey Metapopulation Models,. Theoret. Population Biol..

[17] Cheng KS (1981). Uniqueness of a limit cycle for a predator–prey system. SIAM Journal on Applied Mathematics.

[18] Chicone, C. Ordinary differential equations with applications, (2nd edition), Texts in Applied Mathematics 34, *Springer*, (2006).

[19] González-Olivares E, Ramos-Jiliberto R (2003). Dynamics consequences of prey refuges in a simple model system: more prey, fewer predators and enhanced stability. Ecological Modelling.

[20] Paul, W. *A Second Course in Elementary Differential Equations*, (Dover Publications), Inc., (2004).

[21] Koura YH, Zhang Y, Liu H (2017). Competitive Interaction Model for Online Social Networks’ Users’ Data Forwarding at a Subnet. Mathematical Problems in Engineering.

[22] Bhatti, S. N. & Crowcroft, J. QoS-Sensitive Flows: Issues in IP Packet Handling, 1089-7801/00/2000 IEEE Internet Computing (2000).

[23] Padhye J, Firoiu V, Towsley D, Kurose J (2000). Modeling TCP Reno performance: A simple model and its empirical validation. IEEE/ACM Trans. on Networking.

